# Age-related increase in live-birth rates of first frozen thaw embryo versus first fresh transfer in initial assisted reproductive technology cycles without PGT

**DOI:** 10.1186/s12958-024-01210-0

**Published:** 2024-04-13

**Authors:** Sarah F Wang, David B Seifer

**Affiliations:** Division of Reproductive Endocrinology and Infertility, Department of Obstetrics, Gynecology and Reproductive Sciences, Yale University School of Medicine, New Haven, USA

**Keywords:** Assisted reproductive technology, Frozen thaw embryo transfer, Fresh embryo transfer, Live birth rates, Cumulative live birth rates, Endometrial receptivity

## Abstract

**Background:**

The landscape of assisted reproductive technology (ART) has seen a significant shift towards frozen-thawed embryo transfers (FET) over fresh transfers, driven by technological advancements and clinical considerations. This study aimed to compare live birth outcomes between primary FET and fresh transfers, focusing on cycles without preimplantation genetic testing (PGT), using United States national data from the SART CORS database spanning from 2014 to 2020.

**Methods:**

We performed a retrospective cohort study of autologous first ART cycles without PGT comparing primary embryo transfer (frozen thaw vs. fresh) success rates from the 2014–2020 SARTCORS database. Live-birth rates (LBR) and cumulative live-birth rates (CLBR) were compared between first FET versus first fresh embryo transfer from an index retrieval. Multivariate logistic regression (MLR) determined association between live birth outcomes and method of transfer. In a subsequent sub-analysis, we compared these two embryo transfer methods among patients with either diminished ovarian reserve (DOR) or male factor infertility.

**Results:**

228,171 first ART cycles resulted in primary embryo transfer. 62,100 initial FETs and 166,071 fresh transfers were compared. Initial FETs demonstrated higher LBR and CLBR compared to fresh transfers (LBR 48.3% vs. 39.8%, *p* < 0.001; CLBR 74.0% vs. 60.0%, *p* < 0.0001). MLR indicated greater chances of live birth with FET across all age groups, with adjusted odds ratio (aOR) of live-birth incrementally increasing with advancing age groups. For DOR cycles, LBR and CLBR were significantly higher for FET compared to fresh (33.9% vs. 26.0%, *p* < 0.001, 44.5% vs. 37.6%, *p* < 0.0001), respectively. MF cycles also demonstrated higher LBR and CLBR with FET (52.3% vs. 44.2%, *p* < 0.001, 81.2% vs. 68.9%, *p* < 0.0001), respectively. MLR demonstrated that in DOR cycles, initial FET was associated with greater chance of live birth in age groups ≥ 35yo (*p* < 0.01), with aOR of live birth increasingly considerably for those > 42yo (aOR 2.63, *p* < 0.0001).

**Conclusions:**

Overall LBR and CLBR were greater for first FET than fresh transfers with incremental increases in odds of live birth with advancing age, suggesting the presence of a more favorable age-related change in endometrial receptivity present in frozen-thawed cycles. For both DOR and MF cycles, LBR and CLBR after primary transfer were greater for first FET than fresh. However, this was particularly evident in older ages for DOR cycles. This suggests that supraphysiologic stimulation in older DOR cycles may be detrimental to endometrial receptivity, which is in part corrected for in FET cycles.

## Background

The landscape of assisted reproductive technology (ART) has undergone a significant transformation over the last decade, marked by a substantiative rise in the prevalence of frozen-thawed embryo transfers (FET) in comparison to fresh embryo transfers. This shift, evidenced by an 67.5% increase in global FET cycles between 2010 and 2014, reflects a departure from the conventional role of embryo cryopreservation primarily reserved for surplus embryos or mitigating the risk of ovarian hyperstimulation syndrome (OHSS) [1–3]. This increase in FET cycles can be attributed to the technological advancements in the field of ART, including improvements in vitrification techniques, culture media, and increased use of preimplantation genetic testing (PGT) [1, 4, 5]. Moreover, this increase can also be partially attributed to specific clinical scenarios that necessitate the cancellation of fresh transfers. Studies have shown that fresh embryo transfers are less successful in the presence of conditions such as hydrosalpinges, fluid within the uterine cavity prior to transfer, elevated progesterone levels before retrieval, or the discovery of intrauterine pathologies such as polyps or fibroids prior to the planned fresh transfer [6–9]. However, beyond technological enhancements and clinical considerations, this trend reflects a changing perception of FET in the realm of infertility treatment [4, 5, 10–12]. No longer a mere adjunct to fresh embryo transfer, FET is emerging as a preferable option as more than 75% of treatment cycles in 2020 in the US were FET [13].

The suggested advantages of frozen-thawed embryo transfer (FET) over fresh embryo transfer stem from the rationale that FET facilitates the transfer of embryos into a more physiologically favorable uterine environment. In contrast, the hormonal conditions resulting from ovarian stimulation in fresh in vitro fertilization (IVF) may impact endometrial receptivity, potentially leading to suboptimal embryo implantation and placentation [14–19].

We examined the United States national data from the Society for Assisted Reproductive Technology Clinic Outcome Reporting System (SART CORS) database spanning from 2014 to 2020. The aim of this study was to compare the live birth outcomes associated with primary frozen thawed embryo transfers (FET) and fresh embryo transfers following the initial index retrieval, specifically in cycles without preimplantation genetic testing (PGT).

## Methods

We conducted a retrospective cohort analysis involving autologous first assisted reproductive technology (ART) cycles without preimplantation genetic testing (PGT), to compare the success rates of primary FET versus fresh embryo transfer. The study utilized data from the SART CORS database spanning the years 2014 to 2020.

### Study population

Starting in 2014, SART CORS initiated the practice of linking all transfers, regardless of being fresh or frozen, to their corresponding index retrieval cycles. For this study, we included patients with no prior history of ART who underwent autologous ART and whose initial index retrieval was recorded in the SART CORS database between 2014 and 2020. We excluded patients with a history of prior ART, and cycles involving donor oocytes, embryo banking, gestational carrier or PGT. Only cycles that resulted in embryo transfer were included for analysis. Cycles were stratified based on whether the primary embryo transfer was FET or fresh. The primary embryo transfer was identified as the first transfer for an index retrieval. For FET transfers, this was further defined as the initial transfer occurring within one year of the initial index retrieval.

### Outcome measures

The primary outcomes assessed included the live birth rate (LBR), cumulative live birth rate (CLBR), and miscarriage rates for the first FET compared to the first fresh transfer cycle within the first ART cycle (i.e. first egg retrieval). LBR represented the probability of live birth resulting from the first embryo transfer associated with the initial retrieval. CLBR was defined as the probability of a live birth from all linked embryo transfers within one year from the initial retrieval. Miscarriage rate indicated the probability of pregnancy loss from the first transfer. Patient demographics and primary outcomes were compared with independent sample t-tests and chi-square tests. We performed multiple logistic regression (MLR) analysis to determine the association between live birth outcomes and type of embryo transfer. This analysis was adjusted for age, BMI, nulliparity, race and ethnicity, AMH level, infertility etiology, number of embryos transferred, day 3 and day 5 embryo transfers, use of intracytoplasmic sperm injection (ICSI) and blastocyst transfer.

### Sub-analysis

In a subsequent sub-analysis, we performed a comparative analysis of these two embryo transfer methods within two distinct etiologies of infertility: diminished ovarian reserve (DOR) and male factor infertility. Given that these infertility etiologies are not typically associated with deficiencies in endometrial receptivity, our secondary goal was to investigate the impact of embryo transfer type (fresh versus frozen) on success rates in these specific infertility groups. This aimed to provide a more comprehensive understanding of the efficacy and subtleties surrounding the primary type of embryo transfer.

This sub-analysis focused on patients diagnosed with either DOR or male factor infertility, excluding individuals with both conditions. Patients classified as having DOR or male factor infertility were identified based on the SART CORS etiology of infertility classification. Within this subset of patients, primary outcomes were evaluated to compare live birth rates between primary frozen-thawed embryo transfer (FET) and fresh transfer in initial assisted reproductive technology (ART) cycles among patients with DOR and male infertility, respectively.

### Study data and oversight

The data used for this study were obtained from the SART Clinic Outcome Reporting System (SART CORS). Data were collected through voluntary submission, verified by SART, and reported to the Centers for Disease Control and Prevention (CDC) in compliance with the Fertility Clinic Success Rate and Certification Act of 1992 (Public Law 102–493). SART maintains HIPAA-compliant business associate agreements with reporting clinics. In 2004, following a contract change with the CDC, SART gained access to the SART CORS data system for the purposes of conducting research. Over 90% of all assisted reproductive technology (ART) cycles in the United States are performed at SART-member clinics.

SART annually selects up to 10 clinics, approximately 2.5% of SART clinics, for an on-site validation visit utilizing metrics and a blinded selection process to identify outlier clinics. Medical records are reviewed during the validation visit to verify the designation, outcome, and reporting of cycles. Clinics with significant systematic reporting errors undergo data correction. Six primary metrics and twenty-six secondary metrics are used for clinic selection. The metrics include low prospective reporting for both egg retrieval cycles and total cycles, high live birth rates in the various age groups, low cancellation rate, high percentage of total fertility preservation cycles, high percentage of embryo banking and oocyte banking cycles, high percentage of fertility preservation cycles where oocytes were thawed or embryos were transferred within a year, high percentage of deleted cycles, high percentage of cycles converted from IUI, and low percentage of cycles in which no embryos were suitable for transfer with and without preimplantation genetic testing (PGT). SART does not validate the accuracy of data entry fields such as gonadotropin dosage, number of oocytes retrieved, number of fertilized oocytes, number of embryos cryopreserved, PGT results, or demographic fields such age and diagnosis.

## Results

Between 2014 and 2020, a total of 228,171 women with no prior history of ART underwent an index autologous ART cycle without PGT resulting in embryo transfer. Among these, 62,100 involved primary frozen-thawed FET transfers, while 166,071 involved primary fresh embryo transfers (Table 1). Among primary FET transfers, 5.7% took place on day 3, and the majority (72.4%) occurred on day 5. In comparison, for primary fresh embryo transfers, 23.6% were day 3 transfers, and 65.2% were day 5 transfers. Of note, some transfers fell outside of the day 3 and day 5 categories, with occurrences on days 2, 4, 6, or 7. Consequently, the percentages for day 3 and day 5 transfers do not sum up to 100%. Comparing the demographic and clinical profile between the two groups, women undergoing initial FET were slightly younger at 32.9 years old compared to 34.0 years for those undergoing initial fresh transfers (*p* < 0.0001). Additionally, there was a higher proportion of patients diagnosed with DOR among those undergoing initial fresh transfer compared to FET (20.0% vs. 13.0%, *p* < 0.0001). Women undergoing initial fresh transfers also had a higher mean number of embryos transferred across all age groups compared to their counterparts undergoing FET across all age groups (Table 1, *p* < 0.0001).

**Table 1:** Population demographics and clinical profile by method of primary embryo transfer for 2014–2020 index autologous ART cycles without PGT^a^

Method of Primary Embryo Transfer	FET	Fresh
Number (Patients)	62,100	166,071
Mean age at start (years)	32.9 ± 4.4	34.0 ± 4.5
Race/ethnicity (%)		
White	44.6	45.9
Non-White	55.4	54.1
Asian	9.5	8.3
Black	6.9	5.8
Hispanic	6.4	5.8
Other	7.1	7.4
Unknown	25.5	26.7
Mean D3 FSH (IU/L)^b^	7.3 ± 3.2	7.8 ± 3.8
% Primary Etiology of Infertility^c^		
DOR	13.0	20.0
Tubal	16.1	15.9
Uterine	6.0	4.1
Ovulatory/PCOS	38.1	30.9
Endometriosis	8.6	8.8
Unexplained	14.2	17.8
Male factor	38.3	36.2
Mean number of Embryos Transferred		
< 35	1.3 ± 0.5	1.4 ± 0.5
35–37	1.4 ± 0.5	1.6 ± 0.6
38–40	1.6 ± 0.6	2.0 ± 0.7
41–42	1.8 ± 0.9	2.3 ± 1.0
> 42	2.0 ± 1.2	2.4 ± 1.2
% of Day 5 Embryo Transfers	72.4	65.2

^a^Values are means ± SD

^b^Due to the large number of missing values for AMH, the AMH values were not reported

^c^Multiple diagnoses of infertility were possible; therefore the totals are greater than 100%

The comparison between these two methods of embryo transfers demonstrated significant differences in live birth outcomes. The live birth rate (LBR) for initial FETs was notably higher at 48.3% compared to 39.8% for initial fresh embryo transfers (*p* < 0.001). Similarly, the cumulative live birth rate (CLBR) for initial FETs exhibited a significant increase at 74.0%, in contrast to 60.0% for initial fresh transfers (*p* < 0.0001). Miscarriage rates were slightly greater at 10.5% for FET transfers compared to 7.7% for fresh transfers (*p* < 0.001). Adjustment for patient demographics and clinical profile using multivariate logistic regression (MLR) analysis indicated that initial FETs demonstrated significantly greater adjusted odds ratios (aOR) of live birth across all age groups. Additionally, the aOR for live birth incrementally increased with advancing age (Table 2).

**Table 2:** Adjusted Odds Ratio (aOR) between Primary FET vs. fresh on LBR stratified by age groups

Age (years)	N	Adjusted Odds Ratio (95% CI; p value)
< 35	133,096	1.24 (1.19–1.29, *p* < 0.001)
35–37	47,433	1.24 (1.16–1.33, *p* < 0.001)
38–40	30,211	1.43 (1.31–1.58, *p* < 0.001)
41–42	11,107	1.53 (1.27–1.84, *p* < 0.001)
> 42	6324	1.98 (1.36–2.88, *p* < 0.001)

aOR > 1 implies greater likelihood of LBR with FET

### Diminished ovarian reserve sub-analysis

In the subset of 29,623 women with DOR undergoing their first autologous ART cycle between 2014 and 2020, 5,514 primary FET transfers were compared to 24,109 fresh embryo transfers. The LBR for initial FETs in DOR patients was significantly greater at 33.9%, compared to 26.1% for initial fresh transfers (*p* < 0.001). Similarly, the CLBR for initial FETs in DOR patients was higher at 44.5%, in contrast to 37.6%for initial fresh transfers (*p* < 0.0001). The miscarriage rate was 10.6% for FET transfers and 8.3% for fresh transfers (*p* < 0.001). MLR analysis within the DOR patient group showed a significantly greater chance of live birth for initial FETs, specifically for individuals aged 35 and above, with the adjusted odds ratio (aOR) for live birth showing a substantial increase for those over 42 years (Table 3).

### Male factor infertility sub-analysis

In the sub-analysis involving 65,858 women with male factor infertility, 18,038 initial FETs were compared to 47,820 fresh embryo transfers. The LBR for initial FETs in male factor infertility patients was markedly higher at 52.3%, compared to 44.2% for initial fresh transfers (*p* < 0.001). Likewise, the CLBR for initial FETs was 81.2%, compared to 68.9% initial fresh transfers (*p* < 0.0001). MLR analysis within the male factor infertility patient group demonstrated that initial FETs had a significantly greater chance of live birth in patients who were 40 years or younger (Table 3).

**Table 3:** Adjusted Odds Ratio (aOR) between Primary FET vs. fresh on LBR stratified by age groups for patients with DOR or Male Factor Infertility

Etiology of Infertility	Age (years)	N	Adjusted Odds Ratio (95% CI; p value)
DOR	< 35	7207	1.16 (0.98–1.37), *p* > 0.05
	35–37	6247	1.40 (1.16–1.69), *p* < 0.001
	38–40	7757	1.39 (1.15–1.68), *p* < 0.001
	41–42	4865	1.34 (1.01–1.61), *p* < 0.05
	> 42	3547	2.63 (1.55–4.48), *p* < 0.001
Male Factor	< 35	44,778	1.28 (1.13–1.45), *p* < 0.001
	35–37	13,034	1.33 (1.08–1.63), *p* < 0.01
	38–40	6208	1.57 (1.14–2.16), *p* < 0.01
	41–42	1367	1.22 (0.58–2.59), *p* > 0.05
	> 42	471	0.27 (0.05–1.34), *p* > 0.05

aOR > 1 implies greater likelihood of LBR with FET

## Discussion

In 2014, approximately 40%, or 800,000 cycles of the estimated 2 million annual worldwide ART cycles, were frozen-thawed embryo transfer cycles [3]. By 2020, more than 75% of treatment cycles in the US involved embryo cryopreservation, resulting in more than 200,000 frozen embryo transfers [13]. The observed surge in frozen-thawed embryo transfers (FET) over fresh embryo transfers marks a paradigm shift in the clinical practice of ART over the last 7 years. The rationale behind favoring FET over fresh transfers is, in part, based on the notion that the supraphysiological hormonal levels arising from exogenous gonadotrophins used for ovarian induction during fresh IVF may compromise endometrial receptivity, potentially leading to suboptimal embryo implantation and placentation [1, 5, 14]. Human and animal studies suggest that ovulation induction with exogenous gonadotropins results in endometrial advancement, gene expression changes, and dyssynchronous endometrial stromal and glandular maturation, which may hinder successful implantation [5, 14, 20]. In contrast, in the case of an FET the endometrium is exposed to physiological hormone levels and thus, is more optimal for implantation [1, 5]. Our study delves into this evolving landscape [21–23], comparing the live birth outcomes associated with primary FETs and fresh transfers in cycles without preimplantation genetic testing (PGT).

Our findings demonstrate a clear increase in both overall live birth rate (LBR) and cumulative live birth rate (CLBR) for initial frozen-thawed embryo transfers (FETs) compared to fresh transfers following the first index retrieval. Although miscarriage rates were slightly higher in FET transfers, MLR analysis highlights the significantly greater likelihood of live birth with initial FET across all age groups. Moreover, despite a higher number of embryos transferred in the primary fresh transfer group compared to primary FET, which would typically favor a higher live birth rate (LBR), our analysis revealed a lower LBR for initial fresh transfers compared to initial FETs, underscoring the significance of our findings.

This observed increase in live birth outcomes associated with primary FET remained consistent throughout the range of advancing age, with incremental increases in the odds of live birth as age progressed. This interesting finding suggests that there may be the presence of a more favorable age-related change in endometrial receptivity with frozen-thawed cycles. It implies a potential age-related advantage in utilizing frozen-thawed embryos for primary transfers in older women undergoing ART and potentially augments our evolving understanding of the impact of age on reproductive success [24, 25]. This finding further suggests that there may be age-specific factors at play, where the physiologic conditions associated with frozen-thawed embryo transfers demonstrate a more favorable impact on endometrial receptivity, as women advance in age. If confirmed by additional investigation, utilizing frozen-thawed embryos preferentially to fresh transfer embryos for primary transfers may be more likely to result in a live birth in older women.

Our sub-analyses for individuals with DOR and those with male factor infertility provide additional evidence supporting the advantages of primary FET. In both groups, we observed substantially higher initial LBR following primary FET compared to fresh transfers. Furthermore, the CLBR after primary FET was significantly greater than after fresh transfer for both DOR and male factor infertility patients. These findings underscore the consistent trend of improved outcomes associated with primary FET across diverse patient populations undergoing ART treatment. Moreover, they highlight the potential benefits of prioritizing FET over fresh transfer in specific patient cohorts, reinforcing the importance of tailored treatment approaches to optimize reproductive success.

The age-specific analysis within the DOR patient cohort yielded findings that warrant careful consideration. Among all patients with DOR older than 35, there were consistently higher odds of live birth with FET compared to fresh embryo transfer. In particular, DOR patients age 42 years or older demonstrated the highest adjusted odds ratio (aOR) for live birth compared to all other age groups when undergoing primary frozen-thawed embryo transfer (FET). The intricate interplay between ovarian stimulation protocols and endometrial response, especially in the context of diminished ovarian reserve, has been a subject of ongoing research and debate [14, 15, 26]. The higher aOR for live birth in this age group following primary FET points towards a greater potential vulnerability or diminished endometrial receptivity associated with conventional stimulation protocols in fresh cycles.

This finding suggests a potential negative impact of supraphysiologic stimulation in DOR patients of advanced maternal age, by affecting endometrial receptivity, a phenomenon that is apparently partially mitigated in frozen thawed cycles. These findings suggest that a more individualized approach may be required for this specific older age demographic, whether it be tailored ovarian stimulation or consideration of freeze-all strategies. Further research into the mechanisms underlying this age-dependent variation in outcomes may provide valuable insight for optimizing ART success rates in women with DOR.

Examining the male factor infertility patient subgroup, primary FET was associated with a significantly greater chance of live birth, aligning with the broader trend observed across all patients. However, MLR analysis of this infertility subgroup demonstrated an age-specific advantage. Specifically, patients aged 40 years and younger demonstrated higher likelihood of live birth with primary FET.

These findings underscore the potential benefits of strategically incorporating frozen-thawed embryo transfer depending on the clinical scenario, where FETs exhibit distinct advantages in achieving successful live births. Future research should identify specific demographic populations that stand to benefit from primary FET, contributing to a more personalized and effective approach in assisted reproductive technology.

It is important to acknowledge the limitations of our findings. The retrospective nature of this study, although leveraging a comprehensive uniform and standardized database, precludes causal inferences. Moreover, as the type of transfer was not randomized there may have been untold biases in the selection of patients for fresh or frozen-thawed embryo transfers, potentially impacting the comparability of the groups. Clinicians may have favored transferring the best-quality embryos in fresh cycles, introducing a positive bias toward fresh transfer outcomes. However, despite this potential advantage, the fresh transfer group still demonstrated lower success rates compared to FETs, emphasizing the significance of our findings.

Additionally, it is important to note the higher proportion of patients diagnosed with DOR among women who underwent primary fresh transfer, potentially impacting live birth outcomes. However, even after adjustment for potential confounding variables, including the diagnosis of DOR, our analysis consistently demonstrated a higher likelihood of live birth with frozen-thawed embryo transfers compared to fresh transfers on MLR analysis. By accounting for relevant demographic and clinical characteristics, we ensured that the influence of DOR on outcomes was appropriately controlled for, enabling a more accurate comparison between primary FET and fresh transfers. Notably, this favorable trend towards FET persisted within the DOR subgroup, where initial FETs demonstrated both higher live birth rates and cumulative live birth rates compared to fresh transfers. Moreover, among DOR patients, FETs was associated with a greater chance of live birth for all women aged 35 and older. While we acknowledge the potential impact of DOR on ART outcomes, we also emphasize the multifaceted nature of successful embryo implantation and live birth, which involves factors beyond ovarian reserve alone. Factors such as endometrial receptivity, embryo quality, and treatment protocols also play pivotal roles in achieving successful ART outcomes.

However, the SART CORS database, despite its extensive data, lacks information on specific FET protocols, such as programmed FET versus natural cycles, limiting our ability to factor this into our analysis. The absence of this information prevents a thorough examination of the impact of different FET approaches on ART outcomes. This limitation prevents a comprehensive assessment of the impact of different FET approaches on ART outcomes. Future studies should aim to incorporate detailed information on FET protocols to provide a more comprehensive understanding of their impact on IVF success as well as maternal health.

## Conclusions

These data reveal a consistent advantage in live birth outcomes associated with primary FET across various age groups. The age-specific advantage observed in the DOR infertility cohort underscores the importance of further exploring the possible preferred option of FET in women as they age. Further research to better understand the biological basis of endometrial receptivity being more favorable for a FET than a fresh transfer with advancing maternal age is needed.

## Data Availability

The data that support the findings of this study are available from SART CORS but restrictions apply to the availability of these data, which were used under license for the current study, and so are not publicly available. Data are however available from the authors upon reasonable request and with permission of SART.

## Abbreviations

ART: Assisted Reproductive Technology
BMI: Body Mass Index
CLBR: Cumulative Live Birth Rate
DOR: Diminished Ovarian Reserve
FET: Frozen-Thawed Embryo Transfer
ICSI: Intracytoplasmic Sperm Injection
LBR: Live Birth Rate
MLR: Multivariate Logistic Regression
MF: Male Factor
OHSS: Ovarian Hyperstimulation Syndrome
PGT: Preimplantation Genetic Testing
SART CORS: Society for Assisted Reproductive Technology Clinic Outcome Reporting System
